# Chicago Veterinary Society

**Published:** 1897-05

**Authors:** L. Campbell

**Affiliations:** Secretary


Secretary.

### CHICAGO VETERINARY SOCIETY.

THE meeting was called to order on Thursday evening, April 8th, by the President, Dr. Walker. The minutes of the previous meeting were read and approved. The President made some remarks as to the small attendance, there being only thirteen members present. He requested that the members try to attend earlier, as only ten were present at 9 o'clock, which number is not a quorum. There was no report from the Secretary or Treasurer.

The Telephone Committee did not call on the Telephone Company, owing to the fact that it had come to their knowledge that the company would not consider our application for free telephone use of the slot-machines at drug-stores to call up our own homes and offices, but that the company would consider such an application if made by the druggists. Dr. Walker, of the committee, reported that one druggist had promised to bring up the matter at the next meeting of the druggists.

There were no applications for membership and no unfinished business.

Dr. Gysel, the essayist of the evening, telephoned just previous to the meeting that, owing to the illness of his wife, which was of a serious nature, he would not be able to attend to read his paper on "Operative Treatment of Quittor."

Under new business a letter was read from Dr. John W. Foster offering his resignation to the Society, as he intended leaving Chicago to locate in Springfield, Ohio. Moved and seconded to accept his resignation.

A letter to Dr. A. H. Baker was read from Dr. D. E. Salmon, chairman of the Pasteur Monument Fund. Dr. Baker thought the letter should be considered by the Society. Moved and seconded that, as the communication was not addressed to the Society it be laid on the table, and that the same be returned to Dr. Baker with an explanatory letter.

A very amusing card was shown the members by Dr. Robertson. This card was printed, and was the business card of a would-be veterinary surgeon, who guaranteed to cure, among other affections, CRIBBIN and THISALOE. He also called himself a VETERIANIARY SURGEON. It is a good sample of some of our Western quacks.

Dr. Walker mentioned the fact that our Honorable Governor, Tanner, had appointed for State Veterinarian one Charles P. Lovejoy, of Princeton, Ill., who is a non-graduate and a strong opposer of college qualifications. This appointment was made in the face of strong personal and written opposition to the Governor by a large number of graduated veterinarians throughout the State of Illinois.

On motion, adjourned. L. Campbell,
Secretary.

